# Guanidinium hexa­aqua­zinc(II) bis[tris­(3-carb­oxy­pyridine-2-carboxyl­ato)zincate]

**DOI:** 10.1107/S160053681202987X

**Published:** 2012-07-07

**Authors:** Masoumeh Tabatabaee, Zohreh Derikvand, Jafar Attar Gharamaleki

**Affiliations:** a Department of Chemistry, Yazd Branch, Islamic Azad University, Yazd, Iran; b Department of Chemistry, Faculty of Science, Khorramabad Branch, Islamic Azad University, Khorramabad, Iran; c Young researchers Club, Science and Research Branch, Islamic Azad University, Tehran, Iran

## Abstract

In the title mol­ecular salt, (CH_6_N_3_)_1.30_[Zn(H_2_O)_6_]_0.35_[Zn(C_7_H_4_NO_4_)_3_]_2_, the Zn^II^ atom (site symmetry 3) in the anion is coordinated by three *N*,*O*-bidentate 3-carb­oxy­pyridine-2-carboxyl­ate monoanions to generate a *fac*-ZnN_3_O_3_ octa­hedral coordination geometry. The guanidinium cation (the C atom has site symmetry 3) and the octa­hedral hexa­aqua­zinc(II) dication (the Zn^2+^ cation has site symmetry -3) are occupationally disordered in a 1.30:0.35 ratio. In the crystal, the components are linked by O—H⋯O and N—H⋯O hydrogen bonds to generate infinite (001) sheets. Weak aromatic π–π stacking [centroid–centroid distance = 3.797 (8) Å] is also observed in the crystal.

## Related literature

For related structures, see: Tabatabaee, Abbasi *et al.* (2011 ▶); Tabatabaee, Raza­vimahmoudabadi *et al.* (2011 ▶).
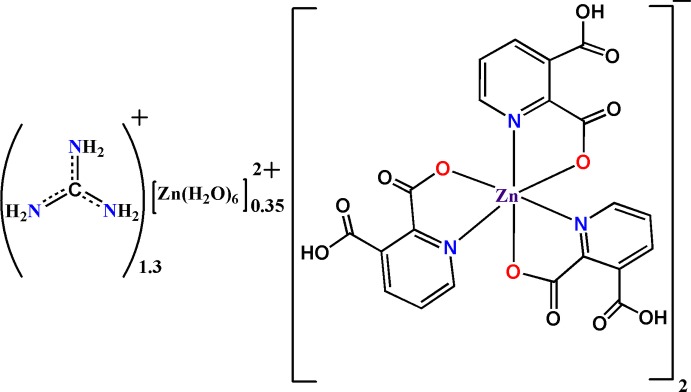


## Experimental

### 

#### Crystal data

(CH_6_N_3_)_1.30_[Zn(H_2_O)_6_]_0.35_[Zn(C_7_H_4_NO_4_)_3_]_2_
*M* *_r_* = 1266.24
Trigonal, 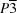

*a* = 14.5775 (16) Å
*c* = 6.3506 (16) Å
*V* = 1168.7 (3) Å^3^
*Z* = 1
Mo *K*α radiation
μ = 1.31 mm^−1^
*T* = 120 K
0.25 × 0.25 × 0.20 mm

#### Data collection

Bruker SMART 1000 CCD diffractometer
Absorption correction: multi-scan (*SADABS*; Bruker, 1998 ▶) *T*_min_ = 0.731, *T*_max_ = 0.773
4304 measured reflections
2079 independent reflections
1443 reflections with *I* > 2σ(*I*)
*R*_int_ = 0.044

#### Refinement

*R*[*F* ^2^ > 2σ(*F* ^2^)] = 0.045
*wR*(*F* ^2^) = 0.106
*S* = 1.00
2079 reflections
129 parameters
H atoms treated by a mixture of independent and constrained refinement
Δρ_max_ = 0.88 e Å^−3^
Δρ_min_ = −0.69 e Å^−3^

### 

Data collection: *SMART* (Bruker, 1998 ▶); cell refinement: *SAINT-Plus* (Bruker, 1998 ▶); data reduction: *SAINT-Plus*; program(s) used to solve structure: *SHELXTL* (Sheldrick, 2008 ▶); program(s) used to refine structure: *SHELXTL*; molecular graphics: *SHELXTL*; software used to prepare material for publication: *SHELXTL*.

## Supplementary Material

Crystal structure: contains datablock(s) I, global. DOI: 10.1107/S160053681202987X/hb6768sup1.cif

Structure factors: contains datablock(s) I. DOI: 10.1107/S160053681202987X/hb6768Isup2.hkl

Additional supplementary materials:  crystallographic information; 3D view; checkCIF report

## Figures and Tables

**Table 1:** Selected bond lengths (Å)

Zn1—O1	2.0818 (18)
Zn1—N1	2.164 (2)
Zn2—O1*S*	2.069 (10)

**Table 2:** Hydrogen-bond geometry (Å, °)

*D*—H⋯*A*	*D*—H	H⋯*A*	*D*⋯*A*	*D*—H⋯*A*
N1*S*—H1*SA*⋯O3^i^	0.86	2.25	3.032 (6)	150
N1*S*—H1*SB*⋯O3	0.86	2.09	2.859 (6)	149
O4—H4*O*⋯O1^ii^	0.85	2.59	3.101 (3)	120
O4—H4*O*⋯O2^ii^	0.85	1.73	2.563 (3)	167

Symmetry codes: (i) 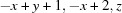; (ii) 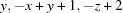.

**Fig. 1.**
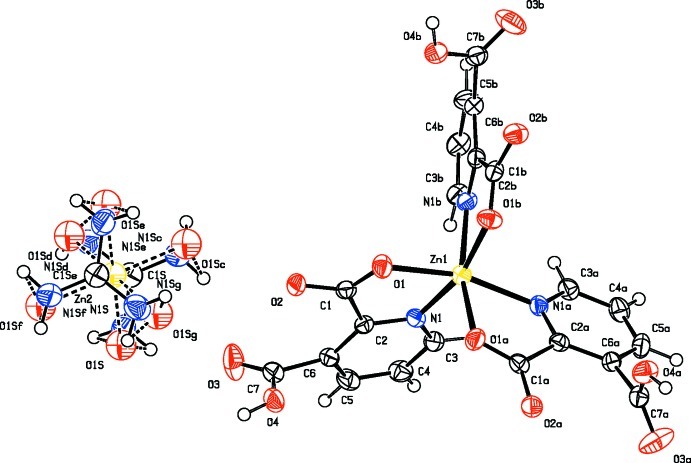
Molecular structure of 1 (displacement ellipsoids drawn at the 50% probability level).

**Fig. 2.**
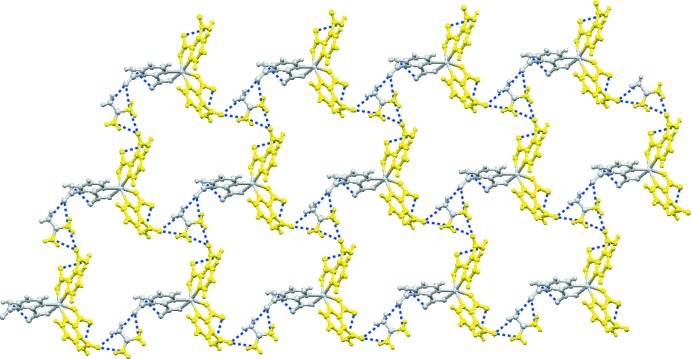
A ball and stick view of the two-dimensional network with showing cavity structure constructed by hydrogen bonds between cationic fragments and anionic complexes.

**Fig. 3.**
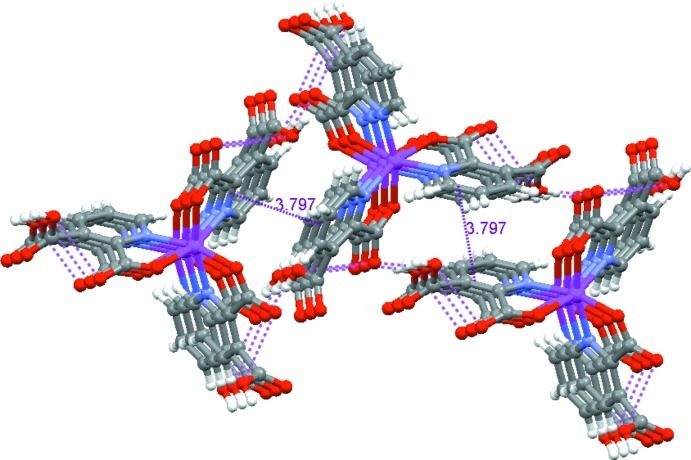
Illustration of polymeric chains formed by O—H···O hydrogen bonds and π-π staking interactions.

## supplementary crystallographic information

### Comment

Unlike pyridine-2,6-dicarboxylic acid, which is an O,N,O-tridentate ligand (e.g. Tabatabaee, Abbasi *et al.*, 2011), pyridine-2,3-dicarboxylic acid often acts as a bidentate chelating ligand through nitrogen and one oxygen atom of the 2-position carboxylic group, while the 3-position carboxylate oxygen atom can act as a bridging atom between metal ions to form a coordination polymer. Recently, we have reported the synthesis and characterization of two one-dimensional coordination polymers of Cd^2+^ and Co^2+^ with pyridine-2,3-dicarboxylic acid (2,3-H_2_pydc) (Tabatabaee, Razavimahmoudabadi *et al.*, 2011) and in this study we wish to report the crystal structure of a mononuclear Zn(II) complex with (2,3-H_2_pydc) in the presence of guanidine hydrochloride.

The title compound consists of [Zn(2,3-Hpydc)_3_]^-^ anions, (GH)^+^ and [Zn(H_2_O)_6_]^2+^ cations (Fig. 1). Two variant of [Zn(2,3-pydcH)_3_]^-^ anions exist in 1 and to balance the charges, one protonated guanidinium cation and [Zn(H_2_O)_6_]^2+^ are present in 1.30: 0.35 ratio. Zn(II) ion in the title compound is six-coordinated by three (2,3-pydcH)^2–^ anions in O,*N*-bidentate fashion and the geometry of the resulting ZnN_3_O_3_ coordination can be described as distorted octahedral. The bond angles around Zn(II) ion involving *trans* pairs of donor atoms are 165.33 (7) and for the *cis* pairs of donor atoms are in the range of 77.27 (7)– 98.26 (8)°. The bond distances Zn–N and Zn–O in are in accordance with those in related structures (Tabatabaee, Razavimahmoudabadi *et al.*, 2011).

There is some hydrogen bonding interactions such as O—H···O, N—H···O between cations and anions. As was shown in figure 2, Hydrogen bonding interactions contribute to the formation of a two dimensional network cavity structure. There is also π-π stacking interactions between the aromatic rings defined by atoms N1/C2 /C6/C5/C4/C3 [symmetry code: 1-*X*,1-Y,1-*Z*; centroid-centroid distance 3.797 (8) Å; the angle between the planes 0°; the perpendicular distance between the planes 3.588 Å; the slippage 1.24 Å]. Ion pairing, hydrogen bonding, π–π stacking and van der Waals interactions are also effective for packing of the crystal structure (Fig 3).

### Experimental

An aqueous solution of pyridine-2,3-dicarboxylic acid (0.10 g, 0.67 mmol)) and Zn(NO_3_)_2_.6H_2_O (0.09 g, 0.3 mmol) was added to an aqueous solution of guanidine hydrochloride (0.08 g, 0.84 mmol) and NaOH (0.04 g, 1 mmol). The reaction mixture was stirred at 25°C for 3 h. Colorless prisms of the title compound were obtained after few days.

### Refinement

The hydrogen atoms of NH groups (water molecules) were found in difference Fourier synthesis. The H(C) atom positions were calculated. All hydrogen atoms were refined in isotropic approximation in riding model with the *U*_iso_(H) parameters equal to 1.2 *U*_eq_(Ci), for methyl groups equal to 1.5 *U*_eq_(Cii), where U(Ci) and U(Cii) are respectively the equivalent thermal parameters of the carbon atoms to which corresponding H atoms are bonded.

### Crystal data

**Table:**

(CH_6_N_3_)_1.30_[Zn(H_2_O)_6_]_0.35_[Zn(C_7_H_4_NO_4_)_3_]_2_	*D*_x_ = 1.799 Mg m^−^^3^
*M**_r_* = 1266.24	Mo *K*α radiation, λ = 0.71073 Å
Trigonal, *P*3	Cell parameters from 524 reflections
Hall symbol: -P 3	θ = 3–30°
*a* = 14.5775 (16) Å	µ = 1.31 mm^−^^1^
*c* = 6.3506 (16) Å	*T* = 120 K
*V* = 1168.7 (3) Å^3^	Prism, colorless
*Z* = 1	0.25 × 0.25 × 0.20 mm
*F*(000) = 644	

### Data collection

**Table:**

Bruker SMART 1000 CCD diffractometer	2079 independent reflections
Radiation source: fine-focus sealed tube	1443 reflections with *I* > 2σ(*I*)
Graphite monochromator	*R*_int_ = 0.044
φ and ω scans	θ_max_ = 29.0°, θ_min_ = 1.6°
Absorption correction: multi-scan (*SADABS*; Bruker, 1998)	*h* = −11→13
*T*_min_ = 0.731, *T*_max_ = 0.773	*k* = −15→19
4304 measured reflections	*l* = −5→8

### Refinement

**Table:**

Refinement on *F*^2^	Primary atom site location: structure-invariant direct methods
Least-squares matrix: full	Secondary atom site location: difference Fourier map
*R*[*F*^2^ > 2σ(*F*^2^)] = 0.045	Hydrogen site location: mixed
*wR*(*F*^2^) = 0.106	H atoms treated by a mixture of independent and constrained refinement
*S* = 1.00	*w* = 1/[σ^2^(*F*_o_^2^) + (0.0393*P*)^2^ + 0.497*P*] where *P* = (*F*_o_^2^ + 2*F*_c_^2^)/3
2079 reflections	(Δ/σ)_max_ < 0.001
129 parameters	Δρ_max_ = 0.88 e Å^−^^3^
0 restraints	Δρ_min_ = −0.69 e Å^−^^3^

### Special details

**Table:**

Geometry. All e.s.d.'s (except the e.s.d. in the dihedral angle between two l.s. planes) are estimated using the full covariance matrix. The cell e.s.d.'s are taken into account individually in the estimation of e.s.d.'s in distances, angles and torsion angles; correlations between e.s.d.'s in cell parameters are only used when they are defined by crystal symmetry. An approximate (isotropic) treatment of cell e.s.d.'s is used for estimating e.s.d.'s involving l.s. planes.
Refinement. Refinement of *F*^2^ against ALL reflections. The weighted *R*-factor *wR* and goodness of fit *S* are based on *F*^2^, conventional *R*-factors *R* are based on *F*, with *F* set to zero for negative *F*^2^. The threshold expression of *F*^2^ > σ(*F*^2^) is used only for calculating *R*-factors(gt) *etc*. and is not relevant to the choice of reflections for refinement. *R*-factors based on *F*^2^ are statistically about twice as large as those based on *F*, and *R*- factors based on ALL data will be even larger.

### Fractional atomic coordinates and isotropic or equivalent isotropic displacement parameters (Å^2^)

**Table:**

	*x*	*y*	*z*	*U*_iso_*/*U*_eq_	Occ. (<1)
Zn1	0.6667	0.3333	0.70631 (8)	0.02632 (17)	
N1	0.65271 (16)	0.45142 (17)	0.5247 (3)	0.0244 (5)	
O2	0.80453 (15)	0.64188 (14)	0.9041 (3)	0.0330 (4)	
O3	0.7557 (2)	0.80730 (16)	0.6260 (4)	0.0559 (7)	
O4	0.63483 (15)	0.69485 (15)	0.8490 (3)	0.0367 (5)	
H4O	0.6365	0.7465	0.9133	0.044*	
O1	0.74921 (14)	0.46921 (14)	0.8890 (3)	0.0308 (4)	
C1	0.7503 (2)	0.5512 (2)	0.8267 (4)	0.0247 (5)	
C2	0.68666 (19)	0.5418 (2)	0.6313 (4)	0.0235 (5)	
C3	0.6077 (2)	0.4408 (2)	0.3371 (4)	0.0278 (6)	
H3A	0.5820	0.3758	0.2631	0.033*	
C4	0.5971 (2)	0.5207 (2)	0.2472 (4)	0.0321 (6)	
H4A	0.5686	0.5128	0.1095	0.039*	
C5	0.6284 (2)	0.6119 (2)	0.3589 (4)	0.0329 (6)	
H5A	0.6206	0.6676	0.3002	0.039*	
C6	0.6715 (2)	0.6227 (2)	0.5585 (4)	0.0249 (5)	
C7	0.6941 (2)	0.7188 (2)	0.6845 (4)	0.0296 (6)	
N1S	0.9175 (4)	1.0138 (4)	0.7528 (8)	0.0448 (11)	0.65
C1S	1.0000	1.0000	0.7526 (11)	0.0393 (18)	0.65
H1SA	0.9332	1.0779	0.7247	0.047*	
H1SB	0.8556	0.9641	0.7169	0.047*	
Zn2	1.0000	1.0000	1.0000	0.0402 (6)*	0.35
O1S	0.8856 (7)	1.0091 (8)	0.8232 (14)	0.057 (3)*	0.35

### Atomic displacement parameters (Å^2^)

**Table:**

	*U*^11^	*U*^22^	*U*^33^	*U*^12^	*U*^13^	*U*^23^
Zn1	0.0236 (2)	0.0236 (2)	0.0317 (3)	0.01181 (11)	0.000	0.000
N1	0.0240 (11)	0.0250 (12)	0.0270 (10)	0.0144 (10)	−0.0022 (8)	−0.0029 (8)
O2	0.0314 (10)	0.0266 (10)	0.0415 (10)	0.0148 (9)	−0.0141 (8)	−0.0112 (8)
O3	0.0712 (17)	0.0240 (12)	0.0586 (14)	0.0133 (12)	0.0215 (12)	0.0021 (10)
O4	0.0365 (12)	0.0287 (11)	0.0448 (11)	0.0161 (9)	0.0097 (9)	−0.0063 (9)
O1	0.0356 (11)	0.0242 (10)	0.0330 (10)	0.0152 (9)	−0.0097 (8)	−0.0006 (7)
C1	0.0204 (13)	0.0282 (14)	0.0262 (12)	0.0125 (11)	0.0000 (9)	−0.0028 (10)
C2	0.0192 (13)	0.0235 (13)	0.0262 (11)	0.0095 (11)	0.0008 (10)	−0.0019 (10)
C3	0.0267 (14)	0.0295 (14)	0.0289 (13)	0.0153 (12)	−0.0049 (10)	−0.0076 (10)
C4	0.0330 (15)	0.0404 (17)	0.0274 (13)	0.0216 (14)	−0.0070 (11)	−0.0027 (11)
C5	0.0371 (16)	0.0323 (16)	0.0376 (14)	0.0236 (14)	−0.0031 (12)	0.0033 (12)
C6	0.0229 (13)	0.0235 (13)	0.0300 (12)	0.0128 (11)	0.0023 (10)	0.0004 (10)
C7	0.0301 (15)	0.0278 (15)	0.0345 (14)	0.0172 (13)	−0.0023 (11)	−0.0009 (11)
N1S	0.039 (3)	0.036 (3)	0.055 (3)	0.015 (2)	−0.010 (2)	−0.001 (2)
C1S	0.038 (3)	0.038 (3)	0.042 (4)	0.0189 (14)	0.000	0.000

### Geometric parameters (Å, º)

**Table:**

Zn1—O1^i^	2.0818 (18)	C4—H4A	0.9500
Zn1—O1	2.0818 (18)	C5—C6	1.388 (4)
Zn1—O1^ii^	2.0818 (18)	C5—H5A	0.9500
Zn1—N1^i^	2.164 (2)	C6—C7	1.500 (4)
Zn1—N1^ii^	2.164 (2)	N1S—C1S	1.314 (5)
Zn1—N1	2.164 (2)	N1S—H1SA	0.8635
N1—C3	1.332 (3)	N1S—H1SB	0.8592
N1—C2	1.337 (3)	C1S—N1S^iii^	1.314 (5)
O2—C1	1.252 (3)	C1S—N1S^iv^	1.314 (5)
O3—C7	1.204 (3)	Zn2—O1S^v^	2.069 (10)
O4—C7	1.288 (3)	Zn2—O1S^iii^	2.069 (10)
O4—H4O	0.8453	Zn2—O1S^vi^	2.069 (10)
O1—C1	1.252 (3)	Zn2—O1S	2.069 (10)
C1—C2	1.514 (3)	Zn2—O1S^vii^	2.069 (10)
C2—C6	1.383 (3)	Zn2—O1S^iv^	2.069 (10)
C3—C4	1.374 (4)	O1S—H1SA	1.0872
C3—H3A	0.9500	O1S—H1SB	0.8897
C4—C5	1.369 (4)		
			
O1^i^—Zn1—O1	91.96 (7)	C4—C5—H5A	120.2
O1^i^—Zn1—O1^ii^	91.96 (7)	C6—C5—H5A	120.2
O1—Zn1—O1^ii^	91.96 (7)	C2—C6—C5	117.7 (2)
O1^i^—Zn1—N1^i^	77.27 (7)	C2—C6—C7	124.3 (2)
O1—Zn1—N1^i^	98.26 (8)	C5—C6—C7	117.9 (2)
O1^ii^—Zn1—N1^i^	165.33 (7)	O3—C7—O4	125.5 (3)
O1^i^—Zn1—N1^ii^	98.26 (8)	O3—C7—C6	122.3 (2)
O1—Zn1—N1^ii^	165.33 (7)	O4—C7—C6	112.1 (2)
O1^ii^—Zn1—N1^ii^	77.27 (7)	C1S—N1S—H1SA	113.5
N1^i^—Zn1—N1^ii^	94.25 (7)	C1S—N1S—H1SB	121.8
O1^i^—Zn1—N1	165.33 (8)	H1SA—N1S—H1SB	117.1
O1—Zn1—N1	77.27 (7)	N1S^iii^—C1S—N1S^iv^	120.000 (7)
O1^ii^—Zn1—N1	98.26 (8)	N1S^iii^—C1S—N1S	120.000 (4)
N1^i^—Zn1—N1	94.25 (7)	N1S^iv^—C1S—N1S	120.000 (11)
N1^ii^—Zn1—N1	94.25 (7)	O1S^v^—Zn2—O1S^iii^	86.7 (3)
C3—N1—C2	119.1 (2)	O1S^v^—Zn2—O1S^vi^	93.3 (3)
C3—N1—Zn1	128.38 (18)	O1S^iii^—Zn2—O1S^vi^	86.7 (3)
C2—N1—Zn1	112.24 (15)	O1S^v^—Zn2—O1S	180.000 (2)
C7—O4—H4O	115.7	O1S^iii^—Zn2—O1S	93.3 (3)
C1—O1—Zn1	117.25 (15)	O1S^vi^—Zn2—O1S	86.7 (3)
O1—C1—O2	125.7 (2)	O1S^v^—Zn2—O1S^vii^	93.3 (3)
O1—C1—C2	117.3 (2)	O1S^iii^—Zn2—O1S^vii^	180.0 (6)
O2—C1—C2	116.9 (2)	O1S^vi^—Zn2—O1S^vii^	93.3 (3)
N1—C2—C6	122.2 (2)	O1S—Zn2—O1S^vii^	86.7 (3)
N1—C2—C1	114.3 (2)	O1S^v^—Zn2—O1S^iv^	86.7 (3)
C6—C2—C1	123.3 (2)	O1S^iii^—Zn2—O1S^iv^	93.3 (3)
N1—C3—C4	122.0 (2)	O1S^vi^—Zn2—O1S^iv^	180.0 (3)
N1—C3—H3A	119.0	O1S—Zn2—O1S^iv^	93.3 (3)
C4—C3—H3A	119.0	O1S^vii^—Zn2—O1S^iv^	86.7 (3)
C5—C4—C3	119.0 (2)	Zn2—O1S—H1SA	102.1
C5—C4—H4A	120.5	Zn2—O1S—H1SB	118.7
C3—C4—H4A	120.5	H1SA—O1S—H1SB	95.5
C4—C5—C6	119.7 (2)		
			
O1^i^—Zn1—N1—C3	133.0 (3)	Zn1—N1—C2—C1	14.2 (3)
O1—Zn1—N1—C3	176.6 (2)	O1—C1—C2—N1	−12.2 (3)
O1^ii^—Zn1—N1—C3	−93.3 (2)	O2—C1—C2—N1	163.7 (2)
N1^i^—Zn1—N1—C3	79.05 (17)	O1—C1—C2—C6	172.7 (2)
N1^ii^—Zn1—N1—C3	−15.5 (2)	O2—C1—C2—C6	−11.4 (4)
O1^i^—Zn1—N1—C2	−53.4 (4)	C2—N1—C3—C4	1.5 (4)
O1—Zn1—N1—C2	−9.84 (16)	Zn1—N1—C3—C4	174.73 (19)
O1^ii^—Zn1—N1—C2	80.30 (17)	N1—C3—C4—C5	−3.8 (4)
N1^i^—Zn1—N1—C2	−107.4 (2)	C3—C4—C5—C6	1.0 (4)
N1^ii^—Zn1—N1—C2	158.04 (18)	N1—C2—C6—C5	−6.1 (4)
O1^i^—Zn1—O1—C1	173.28 (19)	C1—C2—C6—C5	168.6 (2)
O1^ii^—Zn1—O1—C1	−94.7 (2)	N1—C2—C6—C7	171.1 (2)
N1^i^—Zn1—O1—C1	95.86 (19)	C1—C2—C6—C7	−14.2 (4)
N1^ii^—Zn1—O1—C1	−52.4 (4)	C4—C5—C6—C2	3.7 (4)
N1—Zn1—O1—C1	3.33 (18)	C4—C5—C6—C7	−173.7 (3)
Zn1—O1—C1—O2	−172.2 (2)	C2—C6—C7—O3	121.4 (3)
Zn1—O1—C1—C2	3.2 (3)	C5—C6—C7—O3	−61.4 (4)
C3—N1—C2—C6	3.6 (4)	C2—C6—C7—O4	−63.4 (3)
Zn1—N1—C2—C6	−170.68 (19)	C5—C6—C7—O4	113.8 (3)
C3—N1—C2—C1	−171.6 (2)		

Symmetry codes: (i) −*x*+*y*+1, −*x*+1, *z*; (ii) −*y*+1, *x*−*y*, *z*; (iii) −*x*+*y*+1, −*x*+2, *z*; (iv) −*y*+2, *x*−*y*+1, *z*; (v) −*x*+2, −*y*+2, −*z*+2; (vi) *y*, −*x*+*y*+1, −*z*+2; (vii) *x*−*y*+1, *x*, −*z*+2.

### Hydrogen-bond geometry (Å, º)

**Table:**

*D*—H···*A*	*D*—H	H···*A*	*D*···*A*	*D*—H···*A*
N1*S*—H1*SA*···O3^iii^	0.86	2.25	3.032 (6)	150
N1*S*—H1*SB*···O3	0.86	2.09	2.859 (6)	149
O4—H4*O*···O1^vi^	0.85	2.59	3.101 (3)	120
O4—H4*O*···O2^vi^	0.85	1.73	2.563 (3)	167

Symmetry codes: (iii) −*x*+*y*+1, −*x*+2, *z*; (vi) *y*, −*x*+*y*+1, −*z*+2.
